# Impact of an early childhood intervention on the home environment, and subsequent effects on child cognitive and emotional development: A secondary analysis

**DOI:** 10.1371/journal.pone.0219133

**Published:** 2019-07-03

**Authors:** Massimiliano Orri, Sylvana M. Côté, Richard E. Tremblay, Orla Doyle

**Affiliations:** 1 McGill Group for Suicide Studies, Douglas Mental Health University Institute & Department of Psychiatry, McGill University, Montreal, Canada; 2 Bordeaux Population Health Research Centre, INSERM U1219 and University of Bordeaux, Bordeaux, France; 3 School of Public Health, University of Montreal, Canada; 4 School of Public Health, Physiotherapy and Sports Science, University College Dublin, Dublin, Ireland; 5 Departments of Pediatrics and Psychology, University of Montréal, Montreal, Canada; 6 UCD School of Economics & UCD Geary Institute for Public Policy, University College Dublin, Belfield, Dublin, Ireland; University of Rennes 1, FRANCE

## Abstract

The objective of this study was to use secondary data from the Preparing for Life (PFL) trial to test (1) the impact of a prenatal-to-age-five intervention targeting women from a disadvantaged Irish community on the quality of the home environment; (2) whether any identified changes in the home environment explain the positive effects of the PFL program on children’s cognitive and emotional development at school entry which have been identified in previous reports of the PFL trial (ES = .72 and .50, respectively). Pregnant women were randomized into a treatment (home visits, baby massage, and parenting program, n = 115) or control (n = 118) group (trial registration: ISRCTN04631728). The home environment was assessed at 6 months, 1½, and 3 years using the Home Observation for Measurement of the Environment (responsiveness, acceptance, organization, learning material, involvement, variety). Cognitive skills were assessed at 5 years using the British Ability Scales. Emotional problems were teacher-reported at 5 years using the Short Early Development Inventory. Latent growth modeling was used to model changes in the home environment, and mediation analyses to test whether those changes explained children outcomes. Compared to controls, treatment children were exposed to more stimulating environments in terms of learning material (B = -1.62, *p* = 0.036) and environmental variety (B = -1.58, *p* = 0.009) at 6 months, but these differences faded at 3 years. Treatment families were also more likely to accept suboptimal child behaviors without using punishment (acceptance score, B = 1.49, *p* = 0.048) and were more organized at 3 years (B = 1.08, *p* = 0.033). None of the changes mediated children’s outcomes. In conclusion, we found that the program positively impacted different home environment dimensions, but these changes did not account for improvements in children’s outcomes. Exploratory analyses suggest that the impact of improvements in the home environment on child outcomes may be limited to specific groups of children. Limitations of the study include the potential lack of generalizability to other populations, the inability to assess the individual treatment components, and sample size restrictions which precluded a moderated mediation analysis.

## Introduction

The quality and quantity of stimulation and support available in the early home environment is a key predictor of healthy cognitive and socio-emotional development [1–3]. The home environment encompasses both relational factors such as the quality of parenting, including maternal warmth, sensitivity, and responsivity toward the child, as well as material factors such as family organization and the availability of resources and learning materials. The home environment is an important predictor of child development and may account for differences in children’s outcomes [4–8]. The quality of the home environment is often poorer in low socioeconomic status (SES) families [1,9]. This may be attributed to a lack of financial resources, as well as the stress generated by poverty. Low SES often clusters with other negative factors for child development, such as low parental education and poor parenting. Thus, providing support to low SES families to raise the quality of the home environment may be an effective strategy for improving the outcomes of disadvantaged children.

Preparing for life (PFL) was an early childhood intervention program targeting families living in low SES communities of Dublin, Ireland. It aimed to promote children’s development by supporting parents from pregnancy until age 4/5 years through enhancing parental competences and encouraging the provision of a high-quality home environment. The intervention included a home visiting program, a baby massage course, and the Triple P Positive Parenting Program. The efficacy of PFL was demonstrated in previously published studies reporting on this randomized controlled trial by showing moderate-to-large effects on mother-reported behavioral problems [10,11], cognition [11,12], and health problems (asthma, chest infections, health visit) up to 4 years of age [11,13]. In addition, the final outcome report from this trial revealed positive treatment effects on emotional problems (e.g. internalizing problems, treatment group mean score 8.96, SD 1.75; control group mean score 7.73, SD 3.04; *p*<0.05; Cohen’s *d* = 0.50) and cognitive skills (treatment group mean score 97.73, SD 14.37; control group mean score 88.00, SD 12.59; *p*<0.01; Cohen’s *d* = 0.72) [14]. Positive short-term changes in aspects of the home environment (at 1½ years of age) were also previously reported [12]. However, mediation analysis testing whether the identified improvements in child outcomes can be attributed to improvements in the home environment and parenting have not been conducted. This study examined: (1) whether the PFL intervention led to changes in the home environment up until 3 years of age using longitudinal analyses (i.e., modeling changes in the home environment over time using random-effect models), and (2) whether changes in the home environment are explanatory mechanisms through which the intervention impacted child development at 5 years (i.e., mediation analyses). Identifying the processes through which the intervention had an impact will provide important information on the amenability of child outcomes to changes in the home environment, which will help refine future intervention strategies.

First, we hypothesized a positive effect of the PFL program on the home environment, as the program specifically targeted both relational (e.g., by improving adaptive parenting practices) and material (e.g., by teaching the importance of age-adapted learning materials) aspects of the family environment. Second, we hypothesized that the observed treatment effects on children’s development would be mediated by the home environment, given the literature identifying associations between the home environment and child outcomes [4–8].

## Materials and methods

All study procedures were approved by the UCD Human Research Ethics Committee, the Rotunda Hospital Ethics Committee, and the National Maternity Hospital Ethics Committee, and was conducted and reported in conformity with CONSORT guidelines (S1 File). All participants gave written informed consent. Details on study design and outcomes have been published [12,13,15–17]. The trial was registered with the ISRCTN register, (unique identifier ISRCTN04631728—The evaluation of the *Preparing For Life* early childhood intervention programme, http://www.controlled-trials.com/ISRCTN04631728, see the protocol in S2 File). The trial was registered post-recruitment rather than prospectively as the trial developers were not aware of this requirement for community-based behavioral interventions when the trial began in 2008. All data from the PFL trial are publically available for use in the Irish Social Science Data Archive www.issda.com.

### Participants, eligibility, and recruitment

**Fig 1:**
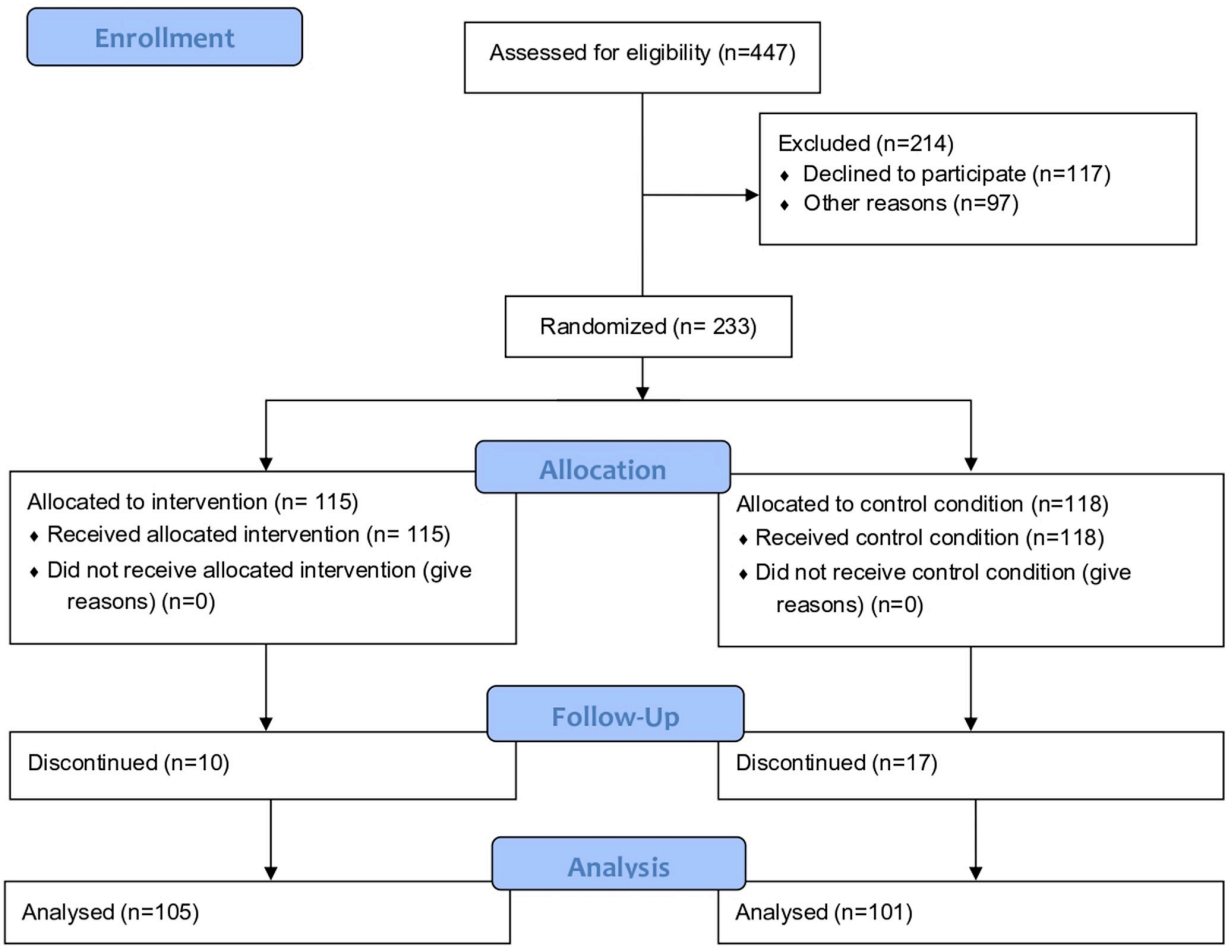
CONSORT flowchart of study participants.

The PFL program was developed as part of the Irish Government’s and The Atlantic Philanthropies’ Prevention and Early Intervention Program (Office of the Minister for Children and Youth Affairs 2008), by 28 local agencies and community groups, with the aim of breaking the intergenerational cycle of disadvantage. The primary outcome of the trial was school readiness. The inclusion criteria included all pregnant women residing in the catchment area between the 29th of January 2008 and the 4th of August 2010. There were no exclusion criteria in order to avoid the stigmatization which may arise with highly selective inclusion criteria. Participation into the program was voluntary and recruitment took place through two maternity hospitals and/or self-referral using a community-based marketing campaign. On average, participants were ~20 weeks pregnant upon recruitment (SD = 7.4 weeks). All participants had to join the program prior to the birth of the child. The program began during pregnancy as evidence shows that maternal health behavior during pregnancy such as diet, as well as maternal stress, can influence children’s later neurodevelopment [18]. A computerized unconditional probability randomization procedure was used to assign 115 and 118 participants to the treatment and control groups respectively (Fig 1). The characteristics of the treatment and control groups at baseline for the estimation sample are presented in Table 1. Statistically significant differences between the groups were found on 9 of 117 baseline measures, which is consistent with chance and indicates the baseline equivalence of the groups [19]. Due to attrition, this study was based on N = 177 participants for which data were available on the variables of interest for at least 1 time point. No evidence of differential attrition between the treatment and control groups was found [20].

**Table 1:** Baseline participant’s characteristics.

	Treatment (N = 86)	Control (N = 91)	*p*-value
Maternal Age	25.6±5.9	25.5±6.1	0.91
First-time Mother %	53%	46%	0.41
Married %	15%	16%	0.97
Low Education % (left school ≤ age 16)	30%	37%	0.34
Unemployed Mothers %	40%	38%	0.99
Resides in Public Housing %	52%	57%	0.62
Long Term Chronic Illness %	8%	14%	0.29
Prior Mental Health Condition %	26%	25%	0.99
Planned Pregnancy %	31%	32%	0.99
Smoked During Pregnancy %	51%	48%	0.82
Drank Alcohol During Pregnancy %	26%	24%	0.69

N = 177. Descriptive statistics are mean (standard deviation) or proportions.

### Treatment and control conditions

The treatment group received 3 supports delivered by mentors with at least a college degree who were specifically hired and trained to implement the PFL program. The mentors received six months training prior to treatment delivery and monthly supervision to ensure fidelity to the program model. During the first five years, the treated parents received fortnightly home visits during which the mentors focused on the identification of developmental milestones and appropriate parenting practices using a curriculum of 210 Tip Sheets covering cognitive development (22 tip sheets), language development (25 tip sheets), approaches to learning (30 tip sheets), socio-emotional development (60 tip sheets), and physical health (105 tip sheets). The Tip Sheets were delivered using role modeling, coaching, discussion, encouragement, and feedback. The treatment group also received 5 two-hour baby massage sessions to promote reciprocal communication between parents and infants and encourage early engagement with the program. When the infants were 2 years old, the treatment group received the Triple P Positive Parenting Program [21]. Triple P is a manualized parenting intervention which aims to improve positive parenting through the use of role play, videos, and vignettes and consists of 5 two-hour group sessions in the local community center, and 3 phone calls encouraging positive parenting practices.

Both the treatment and control groups received some common supports, including a set of developmentally appropriate toys and books, support to participate in community-based social events and public health workshops, newsletters, birthday cards, and access to a support worker who could signpost them to other services. Apart from this, the control group did not receive any additional supports nor receive any additional instructions.

On average, the treatment group received 49.7 (SD = 38.1, range 0–145) home visits between program entry and program end, with an average duration of just under one hour. When the sample is restricted to those who participated in the final assessment, the number of visits was 68. Just under half of the randomized participants took part in some form of the Triple P program and 62 percent attended baby massage.

### Assessment of the home environment

At 6, 18, and 36 months, the Infant-Toddler version of the Home Observation for Measurement of the Environment (HOME) [22] was used to assess the characteristics of the home environment. The HOME assessment focuses on the child as a recipient of inputs from objects, events, and interactions occurring in connection with the home environment. It consists of 45 binary items (0 = false, 1 = true) rated by a trained interviewer using direct observations (18 items, e.g., *“Parent spontaneously vocalises to the child at least twice”*, *“Reading material is present and visible”*), structured interviews with the caregiver (15 items, e.g., “*Tell me about some of the places you go and take the child with you*?”, “*How do you manage meal times at your house*? *Does your child eat at the table with the rest of the family*? *Or do you feed him/her separately*?”), or either of the two (12 items). The interviewers were blinded to participants’ treatment allocation, and were not involved in intervention delivery or data analysis. The HOME assessment ideally took place in the home with the child present and awake. The HOME assesses 6 dimensions of the home environment: Responsiveness (11 items, α = 0.82–0.87), illustrating the degree to which a parent is responsive to the child’s behavior; Acceptance (8 items, α = 0.72–0.80), representing parental acceptance of negative behavior from the child and avoidance of unnecessary punishment; Organization (6 items, α = 0.55–0.70), measuring the degree of routine in a family’s schedule, safety of the environment, and community supports utilized; Learning materials (9 items, α = 0.77–0.86), assessing the appropriateness of play materials for the child; Involvement (6 items, α = 0.56–0.74), illustrating the degree to which the parent is involved in the child’s learning and promotes the child’s development; Variety (5 items, α = 0.42–0.73), assessing visitation of people and attendance of activities that introduce variety into the child’s life. For each dimension, the response for the corresponding items were averaged to obtain the dimension score (high scores indicate a more nurturing home environment). Certain items on the HOME assessment are based purely on interviewer observations of interactions between the parent and the child and of the home environment itself. In the present study, data was missing on a number of HOME subscales as the child was not present and/or the interview did not take place in the home. Specifically, at 6 months, 31% of interviews were conducted outside of the home and/or the child missing, while the corresponding figures at 18 and 36 months were 45% and 47% respectively. Note that the level of missing data was equivalent in the treatment and control groups. As this data may not be missing at random, multiple imputation was performed using STATA’s mi impute command, where missing values were imputed 10 times using the covariates which significantly predicted the probability that the interview was conducted in the home and the probability that the child was present during the interview (additional details are provided in S3 File). Analyses were run with the resulting 10 completed data sets and then pooled with Rubin’s combination rules, using mi estimate.

### Assessment of cognitive and emotional development (study’s primary outcomes)

At 51 months, trained interviewers assessed the children’s cognitive development using the British Ability Scales II: Early Years Battery [23]. The assessment consisted of 6 subscales: verbal comprehension, naming vocabulary, picture similarities, early number concepts, pattern construction, and copying, which were used to derive an overall score reflecting general ability (General Conceptual Ability, CGA) which is centred at 100, with a standard deviation of 15. At 59 months, teachers assessed children’s emotional development using the Short Early Development Inventory (S-EDI), Anxious and Fearful Behavior sub-domain (e.g., “*Would you say that this child comforts a child who is crying or upset*?, α = 0.90) [24]. The S-EDI is composed of 48 core items and provides scores in five domains and 15 subdomains of school readiness. The Anxious and Fearful Behavior subdomain includes 3 items with 3 response options ‘often’, ‘sometimes’, ‘never’.

### Data analysis

**Fig 2:**
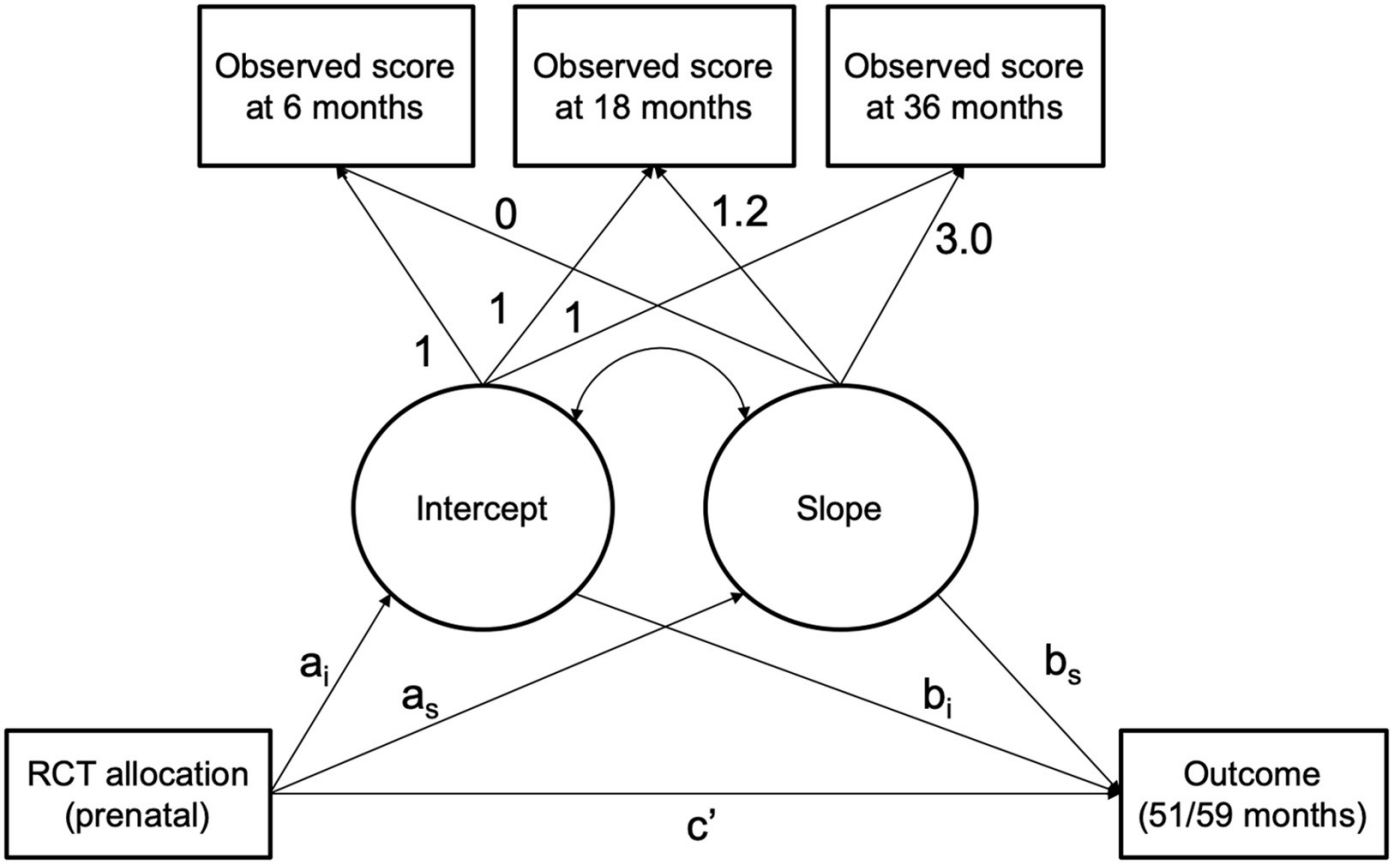
Schematic representation of the hypothesized mediation model. Treatment allocation is a binary variable representing the treatment (coded 1) and control (coded 0) groups. Loadings for the intercept factor were fixed to 1; the first loading for the slope factor was fixed to zero, and the following to 1.2 and 3.6 respecting the laps of time between the observations. The estimated parameter a_i_ and a_s_ represent the effect of the treatment on the initial point (intercept, i.e., 6 months) and on the linear rate of change (i.e., increasing or decreasing) over time of the modeled mediator. The parameters b_i_ and b_s_ represent the effect of the mediator (both intercept and growth factors) on the child outcomes. The estimated parameter ‘c’ represents the direct association, i.e., the remaining non-mediated effect. Estimated residual variances for the observed scores are omitted from the figure for clarity.

Data were analyzed in Mplus 7.4 with Full Information Maximum Likelihood (FIML) [25]. FIML allows the estimation of the parameters even in the presence of incomplete (i.e., missing) data on some variables. Therefore, individuals with at least one data point in any HOME measure or in the outcome were included in the analysis. Estimated models are represented in Fig 2.

#### Trajectories of HOME (mediator)

As a preliminary step, we estimated the trajectories of each of the 6 HOME dimensions using growth models composed of an intercept and a linear slope latent factor (both random effects). All estimated models had a good fit to the data, i.e. non-significant chi-square of model fit, Comparative Fit Indices and Tucker-Lewis indices >0.95, Standardized Root Mean Square Residual and the Root Mean Square Error of Approximation <0.05 and <0.06, respectively [26]. The latent growth model is described using the mathematical equation provided in the S4 File.

#### Treatment effect on HOME trajectories (path a)

The treatment effect on the mediators was estimated by regressing the intercept and slope parameters (*a*_i_ and *a*_s_ in Fig 2) on treatment group membership. First, the effect of the intervention at 6 months was assessed by centering the intercept growth factors (*a*_i_) at 6 months and regressing the intercept on the binary variable indicating the treatment group. Due to the randomized design, any difference between the treatment and control group represents the effect of the intervention on the HOME score at 6 months. To evaluate the effect of the intervention at the subsequent time points, we repeated the procedure by centering the intercept growth factors at 18, then 36 months. Second, the effect of the intervention on the overall rate of change in the HOME dimensions between 6 and 36 months was assessed by regressing the slope parameter (*a*_s_) on the treatment group variable. Participants with data on at least 1 time point were included in the analysis.

#### Effect of HOME on child outcomes (path b)

We estimated the effect of the mediators on child outcomes by regressing the intercept and slope growth factors (*b*_1_ and *b*_s_ in Fig 2) on the outcomes.

#### Indirect (mediation) effect

Finally, we estimated the indirect effect of treatment group membership on child outcomes via the mediators using the distribution of the product approach, i.e., computing unbiased confidence intervals of the a*b product. The mediation model is described using the mathematical equation in the S4 File.

#### Complementary exploratory analyses

Several sets of complementary analyses were conducted. First, mediation analyses were performed using an alternative approach relying on causally-defined indirect effects. Specifically, we used the procedures described by Imai and colleagues [27,28] and implemented in the R package *mediation* [29] to estimate the Average Causal Mediation Effect of the intercept and slope parameters previously estimated. Second, we conducted sensitivity analyses by accounting for omitted mediation-outcome confounding effects by estimating the indirect effect under different degrees of correlation between the mediator and the outcome. Third, we performed mediation analyses using the average of the 6 HOME scales across the 3 time points, owing on the fact that principal component analysis suggested unidimensionality underlying the correlation among these 6 scales. Fourth, exploratory analyses were conducted to test whether any identified mediation effects were stronger for specific groups of children. The hypothesis was that factors identified in the literature as explaining differential responses to interventions (e.g. sex)[30–33] may moderate the indirect effect of the treatment on the outcomes. Thus, we tested the putative moderating role of maternal age (younger than 20 years vs older than 20 years), maternal depressive symptoms (below vs above sample median), and child gender. For each moderator subgroup (e.g., girls and boys) we fitted a mediation model using the average value across the 3 time points of each of the HOME mediators (instead of the growth model) and compared the size of the indirect effect.

The effect size (Hedge’s *g*) of the association was interpreted as follows: ≤ 0.20 = “small”, 0.21–0.50 = “medium”, and >0.50 = “large” effects [34].

As these analyses are based on secondary data from the PFL trial, none were originally planned or published in the trial protocol, thus all analyses should be considered post-hoc. The statistical analysis plan for this paper was not registered prior to analyses. Given the exploratory nature of the study, we did not perform adjustment for multiple testing, following Perneger (1998), Feise (2002), and Rothman, 1990) [35–37].

## Results

### Effect of the intervention on the home environment

**Fig 3:**
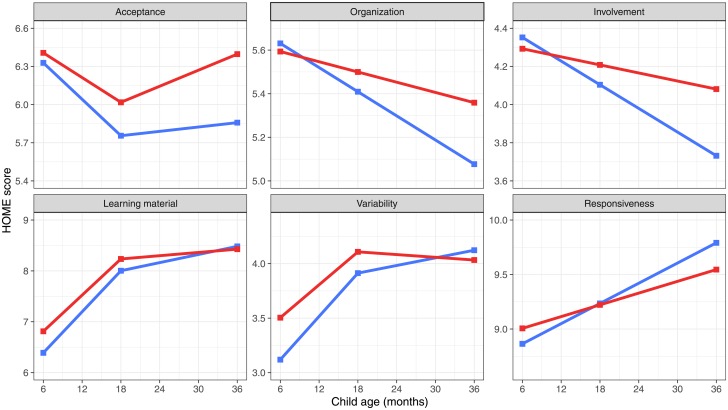
Trajectories of home environment dimensions during early childhood for the treatment and control groups. Blue lines represent the control group, red lines represent the treatment group.

Latent growth models were fitted for 177 participants (86 in the treatment and 91 in the control group). Two main patterns were observed for the HOME dimensions: for the Acceptance, Organization and Involvement dimensions we observed declining trajectories over time indicating dis-improvements in the quality of the home environment, while for the Learning, Variety, and Responsiveness dimensions we observed rising trajectories over time (Fig 3).

For the HOME Acceptance dimension, no initial (6 months) significant difference was found between the treatment and control groups (Table 2). However, the treatment group showed significantly higher scores at 18 (B = 0.25, SE = 0.11, *p* = 0.015) and 36 (B = 0.52, SE = 0.21, *p* = 0.014) months, with increasing effect sizes (i.e., *g* = 0.05, 0.28, and 0.36 at 6, 18, and 36 months, respectively). The decline over time was steeper for the control than the treatment group trajectory (B = 1.49, SE = 0.75, *p* = 0.048, *g* = 0.19). A similar pattern was found for the HOME Organization dimension, also showing declining trajectories for both groups: the group difference at 6 (*g* = -0.06) and 18 (*g* = 0.20) months was not significant, but became significant and of a moderate effect size at 36 months (B = 0.29, SE = 0.11, *p* = 0.011, *g* = 0.37), with the treatment group scoring higher than the control group. The overall slope was also significant (B = 1.08, SE = 0.51, *p* = 0.033, *g* = 0.32). Finally, for the HOME Involvement dimension, we observed a steeper decline for the control than the treatment group trajectories, with moderate effect sizes for the mean differences at 36 months (*g =* 0.22) and on the slope (*g =* 0.21). However, these differences were not significant.

**Table 2:** Treatment effect on the latent growth model parameters.

	Mean (SE) treatment	Mean (SE) control	Effect size	*p-*value
Responsivity				
Intercept at 6 months	9.00 (0.17)	8.95 (0.19)	0.08	0.564
Intercept at 18 months	9.24 (0.13)	9.28 (0.12)	-0.01	0.982
Intercept at 36 months	9.59 (0.14)	9.77 (0.12)	-0.20	0.212
Slope 6–36 months	1.94 (0.69)	2.72 (0.78)	-0.18	0.229
Acceptance				
Intercept at 6 months	5.75 (0.16)	5.71 (0.17)	0.05	0.408
Intercept at 18 months	6.01 (0.09)	5.77 (0.11)	0.28	0.015
Intercept at 36 months	6.39 (0.16)	5.87 (0.17)	0.36	0.014
Slope 6–36 months	2.13 (0.89)	0.55 (0.85)	0.19	0.048
Organization				
Intercept at 6 months	5.59 (0.07)	5.65 (0.07)	-0.06	0.690
Intercept at 18 months	5.50 (0.05)	5.42 (0.05)	0.20	0.169
Intercept at 36 months	5.37 (0.07)	5.09 (0.09)	0.37	0.011
Slope 6–36 months	-0.73 (0.34)	-1.87 (0.38)	0.32	0.033
Learning material				
Intercept at 6 months	8.08 (0.16)	7.74 (0.15)	0.29	0.023
Intercept at 18 months	8.23 (0.09)	8.04 (0.09)	0.27	0.037
Intercept at 36 months	8.45 (0.08)	8.48 (0.06)	-0.08	0.573
Slope 6–36 months	1.22 (0.65)	2.46 (0.58)	-0.27	0.036
Involvement				
Intercept at 6 months	4.27 (0.14)	4.41 (0.12)	-0.05	0.804
Intercept at 18 months	4.19 (0.11)	4.15 (0.1)	0.10	0.432
Intercept at 36 months	4.07 (0.17)	3.78 (0.17)	0.22	0.116
Slope 6–36 months	-0.66 (0.68)	-2.11 (0.67)	0.21	0.144
Variety				
Intercept at 6 months	4.15 (0.15)	3.76 (0.15)	0.28	0.012
Intercept at 18 months	4.11 (0.10)	3.9 (0.11)	0.20	0.124
Intercept at 36 months	4.03 (0.12)	4.13 (0.11)	-0.09	0.560
Slope 6–36 months	-0.4 (0.59)	1.24 (0.56)	-0.03	0.009

The table shows the mean score and standard error (SE) of the HOME dimensions (responsivity, acceptance, organization, learning material, involvement, variety) at each time point (i.e., intercept at 6, 18, and 36 months) and the overall rate of change over time between 6 and 36 months (i.e., slope) for the treatment and control groups. Difference between the treatment and control groups are expressed as effect size (Hedge’s *g*), and the *p*-values for their comparison are based on Wald tests.

Concerning the HOME dimensions demonstrating rising trajectories we found that, for the Learning Materials dimension, the treatment group had significantly higher scores than the control group at 6 (B = 0.43, SE = 0.19, *p* = 0.023, *g* = 0.29) and 18 (B = 0.24, SE = 0.11, *p* = 0.037, *g* = 0.27) months, but not at 36 months (*g* = -0.08). The slopes of the two groups was significantly different (B = -1.62, SE = 0.77, *p* = 0.036, *g* = -0.27), indicating a less steep increase for the treatment group trajectory compared to the control group trajectory. The same was found for the HOME Variety dimension, in which the treatment group showed significantly higher scores than the control group only at 6 months (B = 0.38, SE = 0.15, *p* = 0.012, *g* = 0.28; slope: B = -1.58, SE = 0.60, *p* = 0.009, *g* = -0.03). Finally, for the HOME Responsiveness dimension, no significant differences between treatment and control groups were found.

### Effect of the treatment on children’s cognitive and emotional development mediated through observed changes in the home environment

Mediation analyses are presented in Table 3. For both cognitive and emotional development, the changes observed in the home environment did not explain (mediate) the treatment effect. This is due to nonsignificant associations between the mediators and the outcomes, i.e., few of the b paths were significant.

**Table 3:** Indirect (mediation) effect of treatment on cognitive and emotional development via changes in home environment.

	1.Effect of the treatment on the mediators (a path)		Cognitive development				Emotional development			
			2.Effect of the mediators on the outcome (b path)		3.Mediation effect (a*b)		4.Effect of the mediators on the outcome (b path)		5.Mediation effect (a*b)	
	B (SE)	*p-*value	B (SE)	*p-*value	B (SE)	95%CI	B (SE)	*p-*value	B (SE)	95%CI
Responsivity										
Intercept	0.15 (0.26)	0.564	6.89 (5.46)	0.211	1.03 (-2.42)	-3.23 to 6.88	0.291 (0.63)	0.641	0.04 (-0.20)	-0.34 to 0.54
Slope	-1.28 (1.06)	0.229	0.07 (0.61)	0.905	-0.09 (-1.02)	-2.37 to 2.04	0.00 (0.06)	0.995	0.00 (-0.10)	-0.22 to 0.22
Acceptance										
Intercept	0.08 (0.09)	0.408	8.01 (5.73)	0.162	0.64 (-1.00)	-0.95 to 3.08	0.65 (0.58)	0.266	0.05 (-0.09)	-0.09 to 0.28
Slope	1.49 (0.75)	0.048	3.23 (3.14)	0.305	4.81 (-5.77)	-4.59 to 18.43	0.10 (0.20)	0.636	0.15 (-0.34)	-0.49 to 0.93
Organization										
Intercept	-0.04 (0.09)	0.690	23.88 (52.34)	0.648	-0.96 (-5.58)	-14.34 to 9.84	-4.30 (6.68)	0.520	0.17 (-0.76)	-1.26 to 2.03
Slope	1.08 (0.51)	0.033	-6.49 (8.04)	0.420	-7.01 (-10.16)	-30.51 to 10.73	-0.48 (1.11)	0.666	-0.52 (-1.35)	-3.54 to 2.07
Learning material										
Intercept	0.43 (0.19)	0.023	0.79 (15.69)	0.960	0.34 (-7.38)	-14.94 to 15.88	1.36 (1.26)	0.279	0.58 (-0.65)	-0.49 to 2.09
Slope	-1.62 (0.77)	0.036	-2.00 (4.19)	0.633	3.24 (-7.67)	-11.31 to 20.51	0.19 (0.30)	0.525	-0.31 (-0.56)	-1.58 to 0.71
Involvement										
Intercept	-0.05 (0.19)	0.804	4.17 (2.63)	0.112	-0.21 (-0.95)	-2.35 to 1.68	0.43 (0.41)	0.293	-0.02 (-0.11)	-0.29 to 0.21
Slope	1.39 (0.95)	0.144	1.42 (1.09)	0.191	1.97 (-2.28)	-1.39 to 7.53	0.12 (0.17)	0.486	0.17 (-0.31)	-0.36 to 0.91
Variety										
Intercept	0.38 (0.15)	0.012	3.80 (2.43)	0.117	1.44 (-1.14)	-0.35 to 4.09	-0.13 (0.37)	0.721	-0.05 (-0.15)	-0.38 to 0.25
Slope	-1.58 (0.60)	0.009	1.64 (1.42)	0.248	-2.59 (-2.59)	-8.50 to 1.80	-0.17 (0.20)	0.410	0.27 (-0.35)	-0.36 to 1.06

The table shows (1) the effect (B regression coefficient) of the treatment on the HOME score dimensions at 6 months (ie, intercept) and on the overall rate of change between 6 and 36 months (i.e., slope); see column 1; (2) the effect (B regression coefficient) of the intercept and slope parameters on the cognitive and emotional development outcomes; see columns 2 and 4; (3) the estimated indirect effect of the intercept and slope factor on the cognitive and emotional development outcomes via the mediators (i.e., each HOME dimension); see columns 3 and 5.

### Complementary exploratory analyses

**Fig 4:**
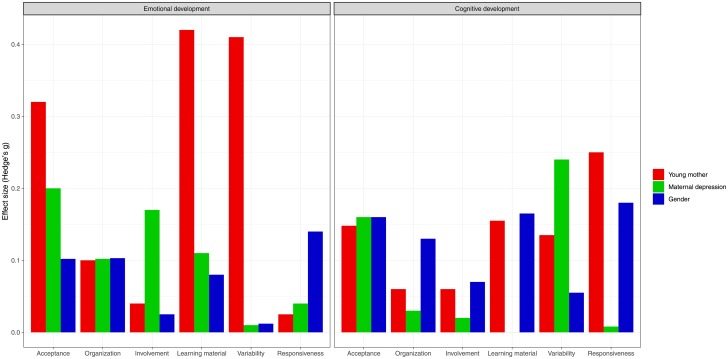
Complementary exploratory analyses. The figure represents the absolute difference in size (y axis; Hedge’s g, >20 = low; 0.20–0.50 = moderate; >0.50 = large) of the indirect effect of each mediator (x axis) between the subgroup defined by the binary moderators (colors), for the 2 outcomes (left, emotional development; right, cognitive development). For example, the indirect effect of variety on internalizing problems is very different for children of younger mothers (< 20 years old) compared to older mothers (> 20 years old).

When we re-estimated our mediation models using causally-defined indirect effects, these analyses (reported in S5 File) were consistent with the primary analyses, showing that none of the Average Causal Mediation Effects reached statistical significance. Additionally, complementary analyses using the mean score of the 6 HOME subscales also did not find any significant mediation effects (S5 File). Sensitivity analyses for those mediation effects indicated that confounding effects of the mediator-outcome association are unlikely to explain the lack of mediation effects (S6 File). To determine if the mediation effects are restricted to subgroups of individuals, we conducted a series of exploratory analyses summarized in Fig 4. The figure represents the difference between the size of the indirect effect for each level of the moderator (e.g., boys) and the size of the indirect effect in the other level (e.g., girls). We observed important differences in effect size of the indirect effect for some of our mediators. Especially, the indirect effect of the treatment on emotional development via acceptance and variety was substantially larger for children of older mothers than for those of younger mothers. Similarly, the indirect effect of the treatment on cognitive skills via responsiveness and learning material was larger for children of older mothers than for those of younger mothers. The indirect effect of the treatment on emotional development via acceptance, and on cognitive skills via variety, was larger for non-depressed mothers than for those of depressed mothers. Test statistics and corresponding *p* values for the mean difference are presented in Fig 4 can be found in the S7 File.

## Discussion

This study demonstrated a positive impact of the PFL intervention on the home environment. However, we did not find that these positive changes in the home environment translated into improvements in children’s cognitive and emotional development at 5 years of age.

We found positive treatment effects on most of the home environment dimensions (acceptance, organization, learning material, and variety), although no effect was found for responsiveness and involvement. This is consistent with findings from previous home visiting programs, where positive impacts on several dimensions of the home environment (*d* = 0.19 from a meta-analysis) [38,39], and parent-child interactions were reported [40–42]. These positive effects on both the relational and material aspects of the home environment are also consistent with the PFL curriculum, which focused on promoting effective parenting (e.g., strategies for dealing with problem behaviors), quality mother-child interactions, and improving knowledge of the needs of the child during different developmental periods (e.g., how to create a safe environment for an infant; how to read to the child; the importance of playing outside; how to support messy play).

In our study, changes in the home environment followed 2 main patterns. First, we observed overall declining scores across early childhood on maternal acceptance, involvement with the child, and organization of family life. Yet the decline was steeper for the control group compared to the treatment group. This suggests that the intervention was effective in maintaining the quality of the home environment in the treatment families in certain domains. It also indicates that the program’s impact may be cumulative i.e., impacting at 1.5 and 3 years, but not at 6 months. Second, we observed an overall increase in the use of learning materials, variability of child activities, and responsiveness to the child across early childhood for both treatment and control groups. For the learning material and variability dimensions, the treatment group had higher scores at 6 months compared to the control group, while at 3 years these differences faded away. This suggests that the efficacy of the intervention in improving the home environment in terms of variability of experience (e.g., offering a wide range of stimulation to the child) and learning materials (e.g., presence of books or appropriate toys) was limited to the early phase of the intervention. Although the differences between the groups were no longer statistically significant at 3 years, treatment children benefited from a more stimulating home environment early in life. This is important as previous studies investigating the impact of the home environment on child development revealed positive associations between early exposure to learning experiences and later language skills [6].

To our knowledge, this is the first study describing the longitudinal development of multiple home environment dimensions over the course of early childhood. The 2 identified patterns are consistent with the home environment’s adaptation to the developing child. For instance, the declining pattern observed for acceptance, organization, and involvement may reflect the home environment’s adaptation to children’s growing autonomy, necessitating less parental involvement. Indeed, as children develop, parents give more autonomy to children in their everyday activities and/or spend less time with their children, many of whom start to attend child care. Also, parents may use more authoritative parenting as children become older and begin to exhibit the normative behavioral problems of toddlerhood [43,44]. This may explain the findings of more acceptance of oppositional child behaviors among treatment parents. Additionally, this pattern may also reflect the difficulty for parents in maintaining the same level of organization as the child become older. Indeed, as children age they are typically involved in more activities and/or a new sibling may have been born, making it more difficult for families to maintain the same level of household organization as when they were parenting an infant. The increasing pattern observed for learning materials, variability, and responsiveness may also reflect the home environment’s adaptation to children’s growing skillset and fields of interest, requiring the environment to be richer in terms of stimulations. For example, more books and activities are required for older than younger children.

The treatment effects on these patterns are also consistent with child development. For example, the larger effect at 3 years than at 6 months for the acceptance dimension, suggests that parents in the treatment group were more prone to accept difficult child behaviors (and thus to use less harsh discipline) when those behaviors started to manifest more frequently. The larger treatment effects at 3 years also suggest that treatment families were able to remain relatively more organized when it became increasingly difficult to do so as the child became more mobile (at 3 years, rather than 6 months). In the same way, the larger effect for learning materials and variety dimensions at 6 months compared to 3 years suggests that parents in the treatment group were able to provide a rich environment in a period during which the importance of providing diversified stimulations is not well known, particularly among low SES families.

However, despite the observed positive treatment effects on most of the home environment dimensions, these effects failed to explain the better cognitive and emotional outcomes among the treatment children which were reported in previous studies. The lack of mediation effects in our analyses is arguably explained by the absence of associations between improvements in the home environment and improvements in child outcomes in our sample (i.e., the b path). Although our small sample size may have limited our power to test mediation, this is unlikely to explain the lack of association between the home environment and child outcomes (i.e., the B paths). This finding is not consistent with other longitudinal studies which typically find a positive relationship between the early home environment and later child outcomes [1–3]. For example, a recent study of mediation effects in an early intervention program in Colombia [45] finds that improvements in material investments (e.g. number of toys and books) were important for children’s cognitive outcomes and improvements in material and time investments (number of times read to the child) were important for socio-emotional outcomes. In our study we only have data on the interactions that took place during the course of the interview.

Another plausible explanation for our lack of mediation effects is the presence of moderation effects which may restrict the positive impact of the improved home environment to specific subgroups of children. Our complementary exploratory analyses suggested that maternal age, maternal depressive symptoms, and child gender may be important moderators to test in future investigations with adequate statistical power. For example, future studies should test the hypotheses that the treatment may particularly improve the home environment of young mothers which subsequently leads to better cognitive outcomes. Due to the lack of mediating association, our hypothesis was not supported and the results call for further investigations into alternative mechanisms explaining the treatment effects on child outcomes.

Another potential explanation for our lack of mediation is the presence of measurement error in the HOME scores. Recent methodological research shows that non-differential measurement error on a mediator may introduce bias in the mediation analysis [46]. However, the impact of measurement error in mediation analysis differs from its impact in a simple regression where it always yields attenuation bias [27]. In mediation analysis it can take unintuitive directions [46]. While it is important to acknowledge its potential influence, in our opinion measurement error is unlikely to completely explain the absence of mediation in our study. To partially address this issue, sensitivity analyses using a factor score of the HOME dimensions (derived from a factor analysis) was used as the mediator. These analyses yielded results consistent with those reported in the main analyses (see S8 File), again suggesting a lack of mediation.

This study has a number of strengths: first, a rigorous trial design, good retention rates, successful randomization, and unlikely contamination [12,13,15–17]. Second, frequent assessment points over early childhood minimizes measurement error and enables longitudinal analyses such as latent growth curve and mediation analyses. Third, the assessment of child outcomes using teacher-reports and direct assessment, as well as assessments of the home environment using observations and interviews, may have minimized misreporting; fourth, the use of random-effect models to describe the development of the home dimensions over time in terms of mean levels, as well as changes over time, and testing indirect effect using the unbiased product-of-the-coefficient approach.

However, the following limitations have to be acknowledged. First, the power analysis was calculated for the primary outcome of the trial, and all analyses reported here are post-hoc analyses. Thus, the tests for moderation effects are likely to be underpowered. The results of this exploratory analyses should be tested in future adequately-powered studies. Second, as mentioned, measurement error may have partially accounted for the lack of significant mediation effects. This possibility should be taken into consideration when interpreting the results. Third, although we conducted sensitivity analyses for mediator-outcome confounding effects, we cannot rule out the possibility that confounding factors may have influenced our results; this may limit any causal interpretation of the mediation analysis. Fourth, the generalizability of our findings is restricted to low SES families. Fifth, we estimated the impact of the full PFL treatment including home visiting, baby massage and Triple P, but it was not possible to disentangle the effect of the individual treatments as PFL is a multi-component program. Sixth, it is important to acknowledge that some of the imputed variables had a high amount of missing data, in particular the HOME Acceptance scale (up to 38% missing data), for which we found a significant effect of the treatment.

In sum, this study found that an early intervention program, delivered to disadvantaged households, was effective in improving the quality of the home environment, however, these improvements did not account for the demonstrated improvements in child outcomes, suggesting that the patterns of treatment effects were mediated through alternative channels or limited to different subgroups of children.

## Supporting information

S1 File CONSORT checklist. (DOC)

S2 File Protocol of the Preparing for Life trial. (PDF)

S3 File Details on missing data in the HOME scales. (DOCX)

S4 File Equations describing the fitted growth and the mediation models. (DOCX)

S5 File Complementary mediation analysis using causally-defined indirect effect and total HOME score. (DOCX)

S6 File Sensitivity analyses for mediation-outcome confounding. (DOCX)

S7 File Test statistics and p-values for the complementary moderated mediation analysis. (DOCX)

S8 File Complementary analysis testing the indirect effect of a factor score of HOME dimensions. (DOCX)
