# Supplementary material for: Impact of an early childhood intervention on the home environment, and subsequent effects on child cognitive and emotional development: A secondary analysis
Source: PLoS One. 2019 Jul 3;14(7):e0219133. doi: 10.1371/journal.pone.0219133 (PMC6608972; doi:10.1371/journal.pone.0219133)
Supplement: S3 File — (DOCX) [file pone.0219133.s003.docx]

**Detail on missing data in the HOME scales**

Certain items on the HOME assessment are based purely on interviewer observations of interactions between the parent and child and of the home environment itself. While the authors of the measure (Caldwell & Bradley, 2003) suggest that the interview should take place in the home with the child present and awake, this was not always possible in the current study and thus there was a substantial level of missing data on some of the HOME items. Specifically, of the 173 interviews conducted at 6 months, 31% were conducted outside of the home with the child missing. At 18 months, 45% of the 154 interviews took place outside the home with the child missing, and at 36 months the corresponding figure was 47% (for a further breakdown see Table 1 below). In these cases, the interviewer could not score the observational items on the HOME assessment as the interview took place in the community centre (at the request of the participant), or the child was asleep or absent during the interview. Table 1 also shows these figures by treatment status and the results of statistical tests of differences between the treatment and control groups regarding whether the child was present at the interview, whether the interview took place outside the home, and the overall level of missing HOME data. As shown, there are no statistically significant differences in the proportion of missing HOME data in the treatment and control groups. Thus, the missing data is balanced across the treatment and control groups.

Due to missing data at the individual item-level, in some cases, a significant number of values were missing for the overall HOME subscales. To exclude these cases from the analyses would reduce the statistical power of the analysis and may bias the estimates of parameters. However, we cannot assume that these observations were missing at random as there may have been an element of self-selection by parents who either did not want the interview to be conducted in the home or did not want their child to be present for the interview. In order to retain the sample and account for self-selection based on observable characteristics, it was necessary to use multiple imputation methods to account for missing data (Rubin, 2003).

This was achieved via a multivariate normal regression approach, which was conducted separately for each time point. If a participant was missing more than half of the items for a particular HOME subscale they were not given an imputed value and they were removed from the analysis. For this reason, some subscales have lower sample sizes than others. Table 2 below detailed the amount of data that was imputed at each time point. As shown, the majority of data were imputed for the HOME Acceptance subscale. This subscale includes items which involve the interviewer observing interactions between the mother and the child. such as whether the mother scolds, shouts or smacks the child during the interview. Therefore, if the child was not present during the interview, these measures could not be taken. However, for the remaining subscales, the amount of missing data imputed is relatively low.

Multiple imputation was performed using STATA’s mi impute command, where missing values were imputed 10 times using the covariates which significantly predicted the probability that the interview was conducted in the home and the probability that the child was present during the interview. To determine which covariates to include in the imputation models, bivariate tests were conducted to determine which of the covariates (measured at the same time as the HOME data) predicted missingness at each time point. The significant covariates were then used in the imputation models. This resulted in different covariates being used in the imputation processes for the 6, 18, and 36 month data. For example, 16 covariates were used to impute the 6 month HOME scores; 23 covariates were used to impute the 18 month HOME scores; and 9 covariates were used to impute the 36 month HOME scores. Treatment status was included as a covariate in all models. Analyses were run with the resulting 10 completed data sets and then pooled with Rubin’s combination rules (Rubin, 1987), using mi estimate. Overall, the mean and dispersion of the imputed and non-imputed data were quite similar. We have amended the manuscript to provide more detail about the imputation process.

**Table 1** Percentage of interviews carried out with the child missing and outside the home by treatment status

|  | **Total interviews complete** | **Child not present during interview** | | **Interview took place outside the home** | | **Interview outside the home and with child missing** | |
| --- | --- | --- | --- | --- | --- | --- | --- |
| **6 Month Interview** | 173 | 25.3% | | 16.5% | | 31% | |
|  |  | Treatment  25.8% | Control 24.7% | Treatment  13.5% | Control  19.8% | Treatment  30.3% | Control 32.1% |
|  |  | chi2=0.03 | p=0.863 | chi2=1.21 | p=0.271 | chi2=0.06 | p=0.804 |
| **18 Month Interview** | 154 | 39.35% | | 20.65% | | 45% | |
|  |  | Treatment  40.0% | Control 38.8% | Treatment 18.7% | Control 22.5% | Treatment 42.7% | Control 47.5% |
|  |  | chi2=0.03 | p=0.873 | chi2=0.35 | p=0.556 | chi2=0.37 | p=0.546 |
| **36 Month Interview** | 149 | 38.67% | | 17.3% | | 47% | |
|  |  | Treatment 36.8% | Control 40.5% | Treatment 18.4% | Control 16.2% | Treatment 46.1% | Control 47.3% |
|  |  | chi2=0.22 | p=0.642 | chi2=0.13 | p=0.721 | chi2=0.02 | p=0.879 |

**Table 2** Amount of data imputed for each HOME scale

| **HOME Scale** | **6 Months** | **18 Months** | **36 Months** |
| --- | --- | --- | --- |
| Responsivity | 9.2% | 9.2% | 4% |
| Acceptance | 26.6% | 37.9% | 38.7% |
| Organisation | 2.3% | 0% | 0.7% |
| Learning Materials | 2.3% | 0% | 1.3% |
| Involvement | 2.9% | 0% | 1.3% |
| Variety | 2.3% | 0% | 0.7% |

**Reference**

Rubin D. Multiple Imputation for Nonresponse in Surveys New York: Wiley 1987.
