# Supplementary material for: Impact of an early childhood intervention on the home environment, and subsequent effects on child cognitive and emotional development: A secondary analysis
Source: PLoS One. 2019 Jul 3;14(7):e0219133. doi: 10.1371/journal.pone.0219133 (PMC6608972; doi:10.1371/journal.pone.0219133)
Supplement: S4 File — (DOCX) [file pone.0219133.s004.docx]

**Equations describing the latent growth model and the mediation analysis**


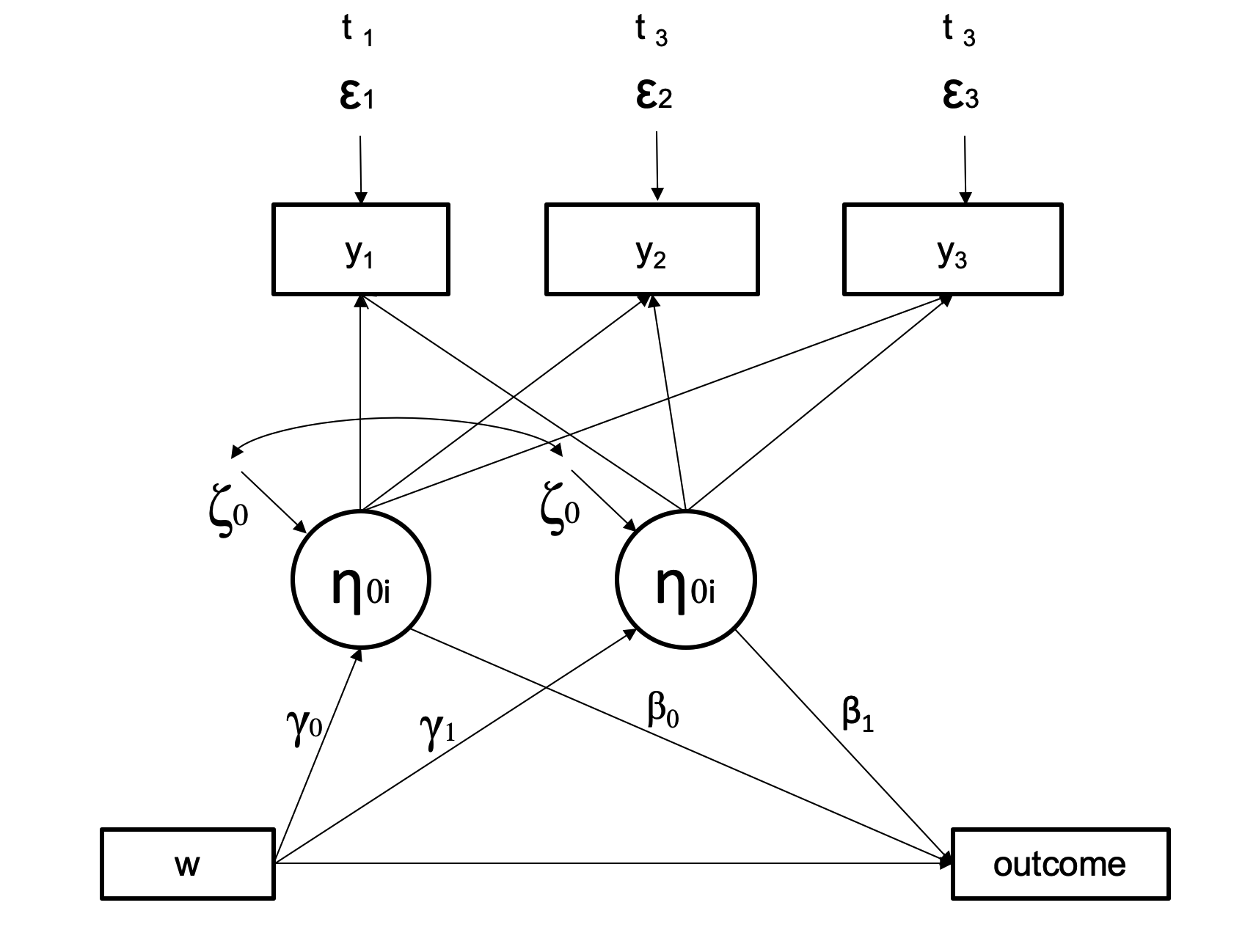


(1) yti = η0i +η1i xt +εti

(2a)  η0i =α0 +γ0 wi +ζ0i

(2b)  η1i =α1+γ1 wi +ζ1i

(c) outcomei = β_0_η0i+ β_1_η1i xt+εi

Where:

t = timepoint. In our case we have 3 time points, so t = {1,2,3}

i = individual

y = outcome of the LGM (ie, modeled variable). In our case y = HOME measure

x = time score. In our case we have the following time scores, x = {0, 1.2, 3.0}: the model was centered at the first point (6 month assumes the time score 0), and the following time points respect the time lag of our measurement. Thus: the second HOME measurement (18 months) is 12 months after the first HOME measurement, so 18 months – 6 months = 12 months, therefore the second time score is 12. The third HOME measurement is at 36 months, which is 30 months after the first one; 36 – 6 = 30, therefore the third time score is 30. We additionally divided by 10 to facilitate model estimation, therefore the final time score where x = {0, 1.2, 3.0}

η0 = intercept

η1 = slope

w = time-invariant covariate. In our case this is the treatment allocation, therefore w = {0,1}

ε, ζ = normally distributed error terms

outcome = the distal outcomes. In our case, this is either the emotional or cognitive development

The indirect effect is therefore calculated from the previous equation using the product of the coefficient approach.

Specifically, we calculated the indirect effect via the intercept as follows:

Indirect_int_ = γ0 × β_0_

And the indirect effect via the slope as follows:

Indirect_slp_ = γ1 × β_1_

It is worth noting that intercept and slope factors are considered together in the same model.
