# Supplementary material for: Impact of an early childhood intervention on the home environment, and subsequent effects on child cognitive and emotional development: A secondary analysis
Source: PLoS One. 2019 Jul 3;14(7):e0219133. doi: 10.1371/journal.pone.0219133 (PMC6608972; doi:10.1371/journal.pone.0219133)
Supplement: S5 File — (DOCX) [file pone.0219133.s005.docx]

**Complementary mediation analysis using causally-defined indirect effect**

The table below show the estimates of the Average Causal Mediation Effect (ACME) and accompanying confidence interval (LCI, lower bound; UCI, upper bound) and *p* value for the two outcomes (i.e., emotional and cognitive development) via the growth factors (intercept and slope) of the growth model, and via the total score of each mediator (i.e., each HOME subscale).

We also reported the mediation effect via the total score of the HOME, both when it is modeled as latent growth curve and when it is average across time point.

|  | 1. Mediation via the Latent Growth Model growth factors (intercept and slope) | | | | | | | | |  | 1. Mediation via the average scores total (6-36 months) | | | |
| --- | --- | --- | --- | --- | --- | --- | --- | --- | --- | --- | --- | --- | --- | --- |
|  | ***Intercept*** | | | |  | ***Slope*** | | | |  |  |  |  |  |
|  | ACME | LCI | UCI | p value |  | ACME | LCI | UCI | p value |  | ACME | LCI | UCI | p value |
| Emotional development |  |  |  |  |  |  |  |  |  |  |  |  |  |  |
| Acceptance | 0.02 | -0.07 | 0.15 | 0.58 |  | 0.03 | -0.12 | 0.20 | 0.59 |  | 0.05 | -0.07 | 0.21 | 0.49 |
| Organization | -0.19 | -0.59 | 0.13 | 0.23 |  | 0.09 | -0.09 | 0.36 | 0.36 |  | -0.06 | -0.23 | 0.04 | 0.32 |
| Involvement | -0.03 | -0.15 | 0.05 | 0.56 |  | 0.02 | -0.13 | 0.18 | 0.82 |  | 0.00 | -0.08 | 0.08 | 0.94 |
| Learning material | 0.04 | -0.14 | 0.31 | 0.76 |  | -0.01 | -0.23 | 0.18 | 0.96 |  | 0.00 | -0.10 | 0.07 | 0.97 |
| Variety | -0.01 | -0.17 | 0.13 | 0.91 |  | 0.06 | -0.11 | 0.28 | 0.53 |  | -0.01 | -0.13 | 0.08 | 0.85 |
| Responsivity | 0.01 | -0.10 | 0.14 | 0.91 |  | 0.01 | -0.11 | 0.18 | 0.89 |  | 0.02 | -0.06 | 0.14 | 0.71 |
| Total score | 0.00 | -0.09 | 0.09 | 0.98 |  | -0.02 | -0.14 | 0.07 | 0.81 |  | -0.03 | -0.16 | 0.06 | 0.58 |
|  |  |  |  |  |  |  |  |  |  |  |  |  |  |  |
| Cognitive development |  |  |  |  |  |  |  |  |  |  |  |  |  |  |
| Acceptance | 0.63 | -0.15 | 1.91 | 0.18 |  | 0.47 | -0.50 | 1.76 | 0.42 |  | 0.90 | -0.01 | 2.42 | 0.07 |
| Organization | 0.54 | -1.99 | 3.23 | 0.66 |  | -0.05 | -2.19 | 2.11 | 0.91 |  | 0.15 | -0.71 | 1.36 | 0.80 |
| Involvement | -0.03 | -0.96 | 0.86 | 0.97 |  | 0.43 | -0.30 | 1.68 | 0.32 |  | 0.17 | -0.55 | 1.18 | 0.65 |
| Learning material | 0.00 | -1.48 | 1.56 | 0.97 |  | 0.53 | -0.60 | 2.53 | 0.44 |  | 0.29 | -0.52 | 1.29 | 0.48 |
| Variety | 0.67 | -0.41 | 2.34 | 0.25 |  | -1.50 | -3.91 | 0.22 | 0.12 |  | 0.39 | -0.33 | 1.52 | 0.34 |
| Responsivity | -0.08 | -1.18 | 0.84 | 0.80 |  | -0.20 | -1.44 | 0.56 | 0.67 |  | -0.07 | -1.16 | 1.04 | 0.87 |
| Total score | 0.19 | -0.45 | 1.08 | 0.62 |  | 0.08 | -0.42 | 0.92 | 0.85 |  | 0.65 | -0.15 | 1.97 | 0.17 |

The table below show the estimates of the Average Causal Mediation Effect (ACME) and accompanying confidence interval (LCI, lower bound; UCI, upper bound) and *p* value for the two outcomes (i.e., emotional and cognitive development) via the total score of the HOME at each time point.

|  | ACME | LCI | UCI | p value |
| --- | --- | --- | --- | --- |
| Emotional development |  |  |  |  |
| Total score at 6 months | 0.00 | -0.14 | 0.16 | 0.98 |
| Total score at 18 months | 0.00 | -0.08 | 0.07 | 0.94 |
| Total score at 36 months | -0.03 | -0.20 | 0.14 | 0.70 |
|  |  |  |  |  |
| Cognitive development |  |  |  |  |
| Total score at 6 months | 0.76 | -0.41 | 2.26 | 0.15 |
| Total score at 18 months | 0.29 | -0.36 | 1.51 | 0.51 |
| Total score at 36 months | 0.10 | -0.50 | 0.89 | 0.68 |
