# Supplementary material for: Impact of an early childhood intervention on the home environment, and subsequent effects on child cognitive and emotional development: A secondary analysis
Source: PLoS One. 2019 Jul 3;14(7):e0219133. doi: 10.1371/journal.pone.0219133 (PMC6608972; doi:10.1371/journal.pone.0219133)

**Sensitivity analysis for potential mediator-outcome confounding effects**

The figures show the Average Causal Mediation Effect (ACME) as a function to the sensitivity parameter rho (ρ). This parameter indicates a range of potential correlations between mediator and outcome introduced by a potential confounding factor. for each value of ρ (x-axis), the 95% confidence intervals (grey area) around the estimated ACMEs virtually always included 0 (i.e., horizontal solid line) and also the estimated ACME assuming no confounding (dotted line, which passes by the origin of the graph).

**Emotional development**


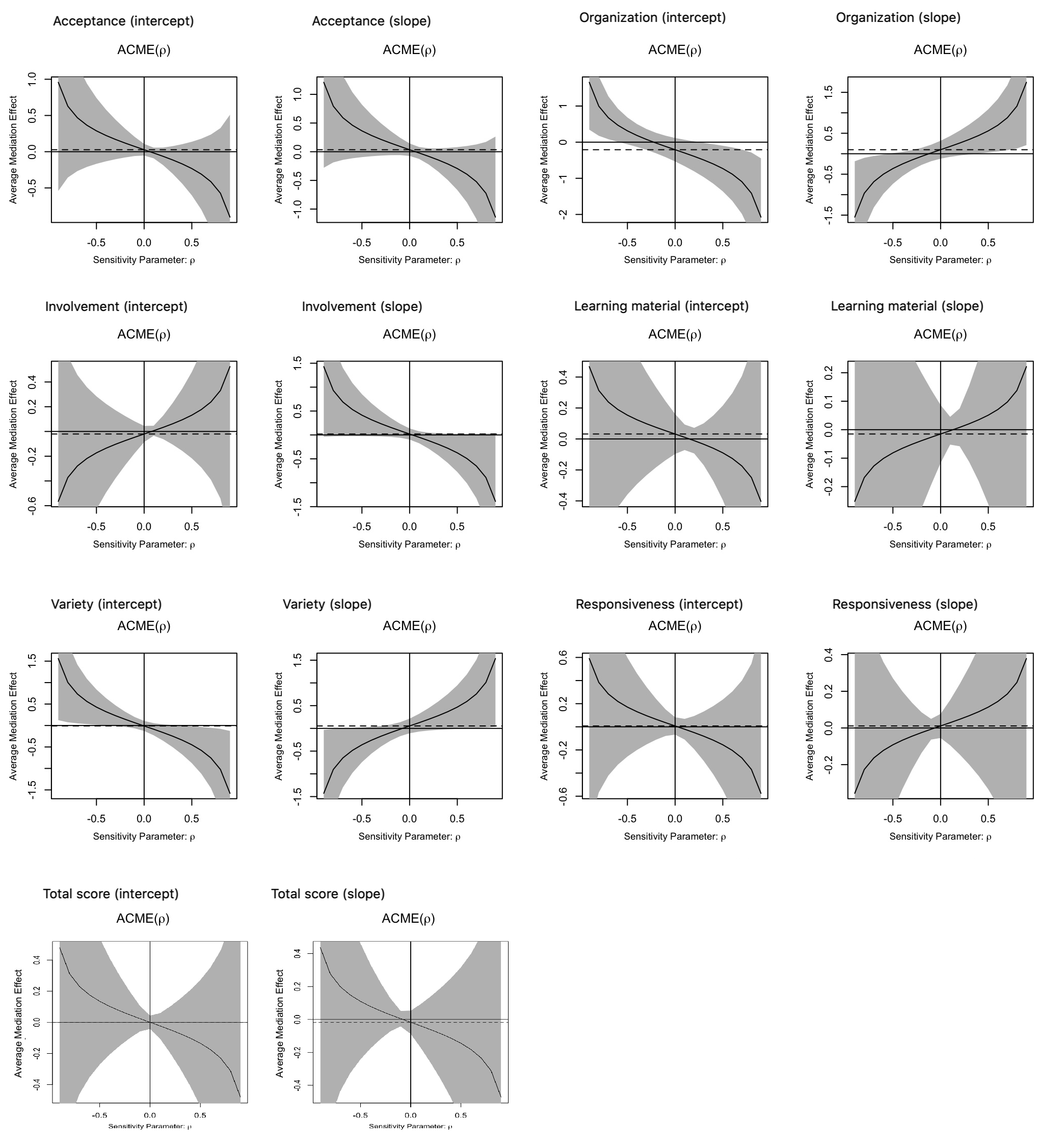


**Cognitive development**


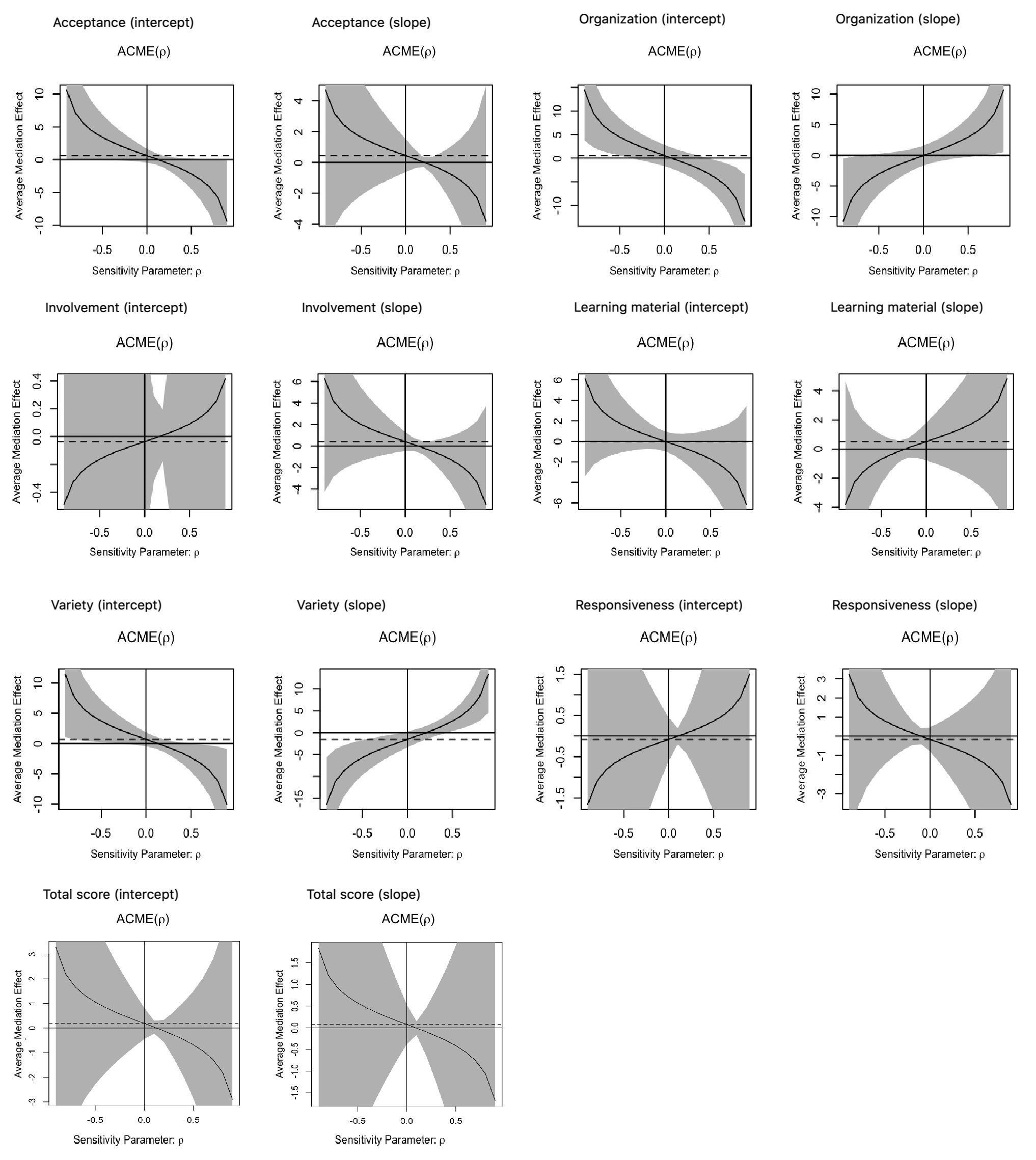

Supplement: S6 File — (DOCX) [file pone.0219133.s006.docx]
