# Supplementary material for: Impact of an early childhood intervention on the home environment, and subsequent effects on child cognitive and emotional development: A secondary analysis
Source: PLoS One. 2019 Jul 3;14(7):e0219133. doi: 10.1371/journal.pone.0219133 (PMC6608972; doi:10.1371/journal.pone.0219133)
Supplement: S7 File — (DOCX) [file pone.0219133.s007.docx]

**Complementary analysis testing the moderation effect of maternal age, maternal depression, and child gender on the indirect effects of the HOME dimensions on our two outcomes**

The table below reports, for each outcome, the mean difference (standardized, i.e., Hedge’s g) between the indirect effects of each HOME scores (columns) of participants with a given moderator factors (first column, i.e., young mother, maternal depression, or gender) and participants without a given moderator factor. The table also show the estimate for the Walt test statistic with 1 DF for the abovementioned mean difference, and the associated *p* value. Results are represented graphically in Fig 2 in the manuscript.

|  | Mediators (HOME dimensions) | | | | | | | | | | | | | | | | | | | | | | |
| --- | --- | --- | --- | --- | --- | --- | --- | --- | --- | --- | --- | --- | --- | --- | --- | --- | --- | --- | --- | --- | --- | --- | --- |
|  | **Acceptance** | | |  | **Organization** | | |  | **Involvement** | | |  | **Learning materials** | | |  | **Variety** | | |  | **Responsivity** | | |
|  | SMD | Wald test | p-value |  | SMD | Wald test | p-value |  | SMD | Wald test | p-value |  | SMD | Wald test | p-value |  | SMD | Wald test | p-value |  | SMD | Wald test | p-value |
| Emotional development |  |  |  |  |  |  |  |  |  |  |  |  |  |  |  |  |  |  |  |  |  |  |  |
| Young mother | 0.32 | 0.48 | 0.49 |  | 0.10 | 0.41 | 0.52 |  | 0.06 | 0.04 | 0.85 |  | 0.16 | 0.72 | 0.40 |  | 0.41 | 0.74 | 0.39 |  | 0.03 | 0.00 | 0.96 |
| Maternal depression | 0.20 | 1.10 | 0.29 |  | 0.10 | 0.47 | 0.50 |  | 0.02 | 0.01 | 0.94 |  | 0.00 | 0.00 | 0.99 |  | 0.01 | 0.00 | 0.97 |  | 0.04 | 0.05 | 0.82 |
| Gender | 0.10 | 0.37 | 0.55 |  | 0.10 | 1.10 | 0.30 |  | 0.07 | 0.19 | 0.67 |  | 0.17 | 1.01 | 0.32 |  | 0.01 | 0.49 | 0.49 |  | 0.14 | 0.75 | 0.39 |
|  |  |  |  |  |  |  |  |  |  |  |  |  |  |  |  |  |  |  |  |  |  |  |  |
| Cognitive development |  |  |  |  |  |  |  |  |  |  |  |  |  |  |  |  |  |  |  |  |  |  |  |
| Young mother | 0.15 | 0.16 | 0.69 |  | 0.06 | 0.06 | 0.81 |  | 0.04 | 0.08 | 0.78 |  | 0.42 | 0.80 | 0.37 |  | 0.14 | 0.34 | 0.56 |  | 0.25 | 0.22 | 0.64 |
| Maternal depression | 0.16 | 0.79 | 0.37 |  | 0.03 | 0.04 | 0.84 |  | 0.17 | 0.90 | 0.34 |  | 0.11 | 0.34 | 0.56 |  | 0.24 | 1.65 | 0.20 |  | 0.01 | 0.00 | 0.98 |
| Gender | 0.16 | 0.95 | 0.33 |  | 0.13 | 0.54 | 0.46 |  | 0.03 | 0.06 | 0.80 |  | 0.08 | 0.00 | 0.96 |  | 0.06 | 0.34 | 0.56 |  | 0.18 | 1.58 | 0.21 |
