# Supplementary material for: Impact of an early childhood intervention on the home environment, and subsequent effects on child cognitive and emotional development: A secondary analysis
Source: PLoS One. 2019 Jul 3;14(7):e0219133. doi: 10.1371/journal.pone.0219133 (PMC6608972; doi:10.1371/journal.pone.0219133)

**Complementary analysis testing the indirect effect of a factor score of HOME dimensions**

The table below reports, for each outcome, the Average Causal Mediation Effect (ACME) of mediation models using a factor score to represent the 6 HOME dimensions (averaged across the 3 time points).

The 6 HOME dimensions were represented with one single factor score as we found that one dimension explain the covariance among these dimensions (unidimensionslity, see scree plot below)

|  | ACME | LCI | UCI | p value |
| --- | --- | --- | --- | --- |
| Emotional development |  |  |  |  |
| HOME factor score | -0.01 | -0.08 | 0.05 | 0.80 |
|  |  |  |  |  |
| Cognitive development |  |  |  |  |
| HOME factor score | 0.59 | -0.10 | 1.17 | 0.14 |


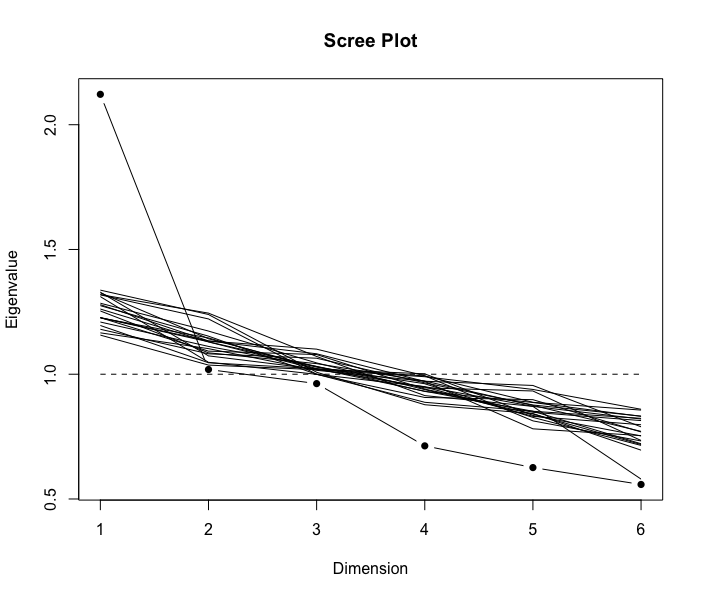

Supplement: S8 File — (DOCX) [file pone.0219133.s008.docx]
